# Multisensory stimuli and pain perception in the newborn

**DOI:** 10.1038/s41390-023-02833-6

**Published:** 2023-10-13

**Authors:** Petra S. Hüppi, Manuela Filippa

**Affiliations:** 1 Division of Development and Growth, Department of Pediatrics, Obstetrics & Gynecology, University of Geneva, Rue Willy-Donzé, Geneva, 1211 Switzerland; 2 Swiss Center for Affective Sciences, Department of Psychology and Educational Sciences, University of Geneva, Boulevard Carl-Vogt 101, Geneva, Switzerland

The study by Anbalagan et al. in this issue of Pediatric Research illustrates the potential role of recorded music for pain relief during minor painful procedures in fullterm newborns, i.e. heel pricks. It adds to the increasing evidence that sensory stimuli during a pain-inducing procedure can reduce behavioral response to pain, here including newborns from a non-white multiethnic social context.

Following the World Health Organization’s summary notes on vaccinations, suggesting that “there are effective, feasible, non-costly, culturally acceptable, and age-specific evidence-based strategies to mitigate pain at the time of vaccination” in infants,^1^ the researchers investigated the analgesic efficacy of a feasible, cost-effective, and non-harmful strategy for protecting newborns from the well-known short effects of pain exposure.

A recent Cochrane review summarizes many non-pharmacological managements of pain relief that have been investigated, they include sensorimotor stimuli through swaddling, rocking, touch, massage, facilitated tucking, sucking with or without sweetener, modulation of unisensory stimuli through sound reduction or sound addition, smell addition, light reduction or multisensory bundles. Sixty-three studies are reported that used the heel prick as standard painful procedure similar to what was used in the current study and represents a fairly standardized event associated with pain and, therefore, ideal to assess pain response and pain regulation from a behavioral response level.^2^ Sound addition showed more effects than sound reduction, which showed no effect on pain reactivity or immediate pain regulation, but the Cochrane review with the data available (including no specific music studies and only two studies one on maternal voice and the other on white noise) concludes that *based on very low-certainty evidence, two studies reported that sound addition was efficacious at reducing pain reactivity in fullterm neonates, but overall the review concludes that the intervention based on the most substantial and least heterogeneous body of literature appears to be non-nutritive sucking. Although this intervention seems to show promise for reducing pain behaviors in full-term newborns (large effect sizes), the evidence suggests very low certainty in the findings.*^3^ While earlier systematic reviews did only report trends on pain reduction by music interventions, a recent meta-analysis by Ting et al. including 38 studies in neonatal and pediatric study populations did show that Music interventions during minor painful procedures did reduce pain with moderate to large effect sizes, but with a mix of painful procedures and different music interventions (classical music, kids’ music, world music, pop music, special composition, multiple combinations).^4^ The authors of the current study, therefore, aimed at designing a blinded well controlled single center clinical trial using recorded music during a standardized heel-prick procedure that includes sucrose administration to the baby prior to the painful event. The music intervention was started 20 min prior and lasted 5 min beyond the painful event, which is a longer period compared to earlier studies^5^ (10 min). The music intervention was given by sound speakers at the vertex of the head of the baby allowing binaural sound exposure but did not allow to blind the intervention to the care team. The assessor, on the other hand, entered the room with sound canceling headphones and different music playing during the assessment of the NIPS, a purely behavioral assessment of pain reactivity and pain regulation based on facial expression, crying, breathing patterns, limb movements, and arousal. Other studies have used headphones for the newborns^6^ that would have allowed to completely blind the caregiver and the assessor team to the music or control intervention. The study deliberately avoided concomitant cuddling or pacifier use during the procedure, to decrease potential bias and both groups received sucrose and the heel prick was performed on day 2 of life without prior painful procedures in the newborn. The authors of the present study did not reach the planned recruitment of 200 newborns because of interruption of the study due to the COVID pandemic, but with 100 participants enrolled this was a rather larger study than prior interventions studies, somewhat accounting for inter-individual pain processing in the sample. The babies in the intervention group were listening to “Deep sleep” from Mozart’s bedtime lullabies for babies during the painful procedure. Even though the trial was conducted in a multiethnic context, where the chosen extract was likely unfamiliar to all participants, the researchers’ choice of music imposes a reflection. The authors thus propose the existence of a general and universal “Mozart effect,” which has been widely discussed, particularly in clinical practice:^7^ which acoustical features could generate specific effects and what role familiarity plays in music selection are questions that remain unanswered. The results show a clear beneficial effect of the music intervention on the NIP scores both at the time of heel prick as well as after the painful procedure with lower mean scores at the event, so lower pain reactivity and lower scores during pain regulation up to 5 min post painful event. It is noted that compared to other studies testing the efficacy of sucrose to reduce pain to heel pricks the current study’s control (sucrose only) group had relatively high NIPS (6–7) and the music intervention group had NIPS similar to other studies that only used sucrose as an intervention, posing the problem of variability in the control conditions in different studies.^8^ This pragmatic trial though makes a point on the role of multisensory stimulation that modulates pain reactivity and pain regulation. An earlier study using different classical music pieces and heart-beat had found similar results with reduced NIPS for all sensory-supported interventions.^9^

However, infant’s pain perception should be investigated from multiple perspectives, involving behavioral, physiological and brain methods, as noxious-evoked brain activity in infants is not always coordinated with behavioral pain expression.^10,11^

So, how can sensory stimulation alter pain perception, and what do we know about neural processing of pain and sensory stimulation? Pain pathways in the brain have been studied by fMRI and classical brain regions active in pain perception are the anterior insula, the middle cingulate cortex, and the thalamus^12^ but perception of pain or no pain is modulated by the pain modulatory system including descending projections from insular and prefrontal cortices via the brainstem periaqueductal gray to the spinal cord and studies have shown that higher connectivity of the anterior insular cortex with the periaqueductal gray is responsible for no-pain perception.^13^ This has recently been replicated by an elegant study on newborn pain modulation showing that increased functional connectivity in the descending pain modulatory system including anterior insula, anterior cingulate, middle frontal gyrus and periaqueductal gray attenuated functional response in the pain cortical areas and so dampen the magnitude of their brain activity in response to incoming nociceptive input.^14^ So, how can music affect functional connectivity in the brain of newborns? Could there be a link to the networks involved in pain perception? We have recently studied extensively the effects of music on functional connectivity in the newborn period and could show that indeed music strengthens connectivity with the salience network composed of the anterior insula and anterior cingulate,^6,15,16^ so the same networks involved in the pain modulatory system. So, it is possible that an increased functional connectivity induced in this network by music listening could impact pain perception.

We also recently demonstrated that live maternal voice administration decreases pain perception in preterm newborns, by increasing oxytocin levels both in infants^17^ and in mothers, who, in turn, decrease their anxiety levels.^18^

Thus, music and maternal voice, singing and speaking, could act both at dampening stress-related biomechanisms, regulating HPA activity and oxytocin production, and at activating an emotional and affective brain response, with a concomitant impact on noxious-evoked brain activity.^19^

Pain relief is a universal right that should be pursued especially for vulnerable and at-risk populations.^20^ The long-term impact of continuous noxious stimuli, particularly in conditions of sensory and affective deprivation, necessitates systematic research on non-pharmacological pain protection studies involving an array of brain, physiological, and behavioral investigation methods.
